# Radiologic zebra line sign in a patient with Langerhans cell histiocytosis on bisphosphonate therapy

**DOI:** 10.1002/jha2.452

**Published:** 2022-04-24

**Authors:** Kenichi Sakamoto, Yoko Shioda

**Affiliations:** ^1^ Department of Pediatrics Shiga University of Medical Science Otsu Japan; ^2^ Children's Cancer Center National Center for Child Health and Development Tokyo Japan

1

A 3‐year‐old boy was diagnosed with multi‐system Langerhans cell histiocytosis (LCH) with bone, skin, lung, lymph node, and bone marrow involvement at 8 months of age. He achieved non‐active disease (NAD) after chemotherapy with prednisolone, vincristine, and cytarabine. However, the patient presented with bone lesion relapse. Despite re‐initiation of vinblastine and prednisolone, his bone lesion progressed, prompting treatment with three cycles of cladribine. While it provided some improvement, he presented with bone lesion relapse soon after cladribine therapy. Due to the recurrence of the bone lesion, we administered bisphosphonate therapy using monthly zoledronic acid for 6 months in addition to conventional chemotherapy, which resolved his bone lesion. The patient achieved NAD for 1 year. While he did not present with bone pain, radiographs obtained during bisphosphonate therapy revealed a “dense metaphyseal band sign,” also known as “zebra line sign,” at the tibia and fibula (Figure 1A−D). Nevertheless, 2 years after initiation of bisphosphonate therapy, his growth remained steady (Figure 1E).

**FIGURE 1:**
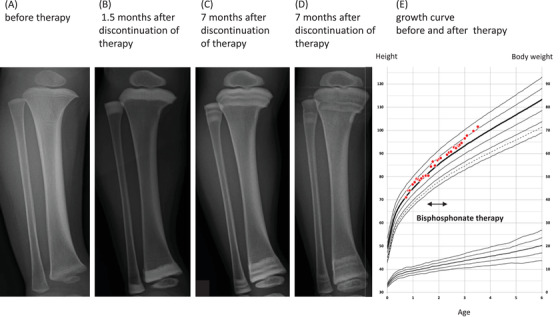
(A−D) “Dense metaphyseal band sign,” also known as “zebra line sign,” at the tibia and fibula. (E) Two years after initiation of bisphosphonate therapy, his growth remained steady

LCH is characterized as both neoplastic and inflammatory disease. Osteoclasts are crucial in the pathogenesis of LCH, especially in lytic bone lesions. Previous reports revealed that bisphosphonate therapy, including pamidronate and zoledronate, was effective in patients with LCH. Bisphosphonates reduce the activity of osteoclasts by inhibiting their recruitment, thereby resulting in the uncoupling of bone remodeling. The zebra line sign refers to the line of cartilage calcification persisting from the metaphysis into the diaphysis [1, 2]. This radiologic finding reflects the administration of bisphosphonate [3]. Although the zebra line sign seems to be a normal reaction to bisphosphonate therapy, hematology‐oncology specialists are not familiar with this finding. In addition, the long‐term side effects (e.g., short stature and fracture) remain unknown. This study highlights the importance of long‐term follow‐up to evaluate the safety profile of bisphosphonates in patients with LCH.

## CONFLICT OF INTEREST

The authors declare no conflict of interest.
